# Oleandrin and Its Derivative Odoroside A, Both Cardiac Glycosides, Exhibit Anticancer Effects by Inhibiting Invasion via Suppressing the STAT-3 Signaling Pathway

**DOI:** 10.3390/ijms19113350

**Published:** 2018-10-26

**Authors:** Young Shin Ko, Trojan Rugira, Hana Jin, Sang Won Park, Hye Jung Kim

**Affiliations:** 1 Department of Pharmacology, College of Medicine, Institute of Health Sciences, Gyeongsang National University, Jinju 52727, Korea; shini33@naver.com (Y.S.K.); rugirawacu@gmail.com (T.R.); hahaha-_-0001@hanmail.net (H.J.); parksw@gnu.ac.kr (S.W.P.); 2 Department of Convergence Medical Science (BK21 Plus), Gyeongsang National University, Jinju 52727, Korea

**Keywords:** breast cancer cells, invasion, oleandrin, odoroside A, STAT-3

## Abstract

The cardiac glycosides oleandrin and odoroside A, polyphenolic monomer compounds extracted from *Nerium oleander*, have been found to have antitumor effects on various tumors at low doses. However, the mechanisms of anticancer effects of oleandrin and odoroside A are not well known. Therefore, in this study, we aimed to investigate the anticancer effects of oleandrin and odoroside A and their associated mechanisms in highly metastatic MDA-MB-231 breast cancer cells and radiotherapy-resistant (RT-R) MDA-MB-231 cells. Our results showed that oleandrin and odoroside A dose-dependently decreased the colony formation and the invasion of both cell lines at nanomolar ranges. Furthermore, oleandrin (50 nM) and odoroside A (100 nM) reduced octamer-binding transcription factor 3/4 (OCT3/4) and β-catenin levels and matrix metalloproteinase-9 (MMP-9) activity. Finally, we found that phospho-STAT-3 levels were increased in MDA-MB-231 and RT-R-MDA-MB-231, but not in endothelial cells (ECs), and that the levels were significantly decreased by oleandrin (50 nM) and odoroside A (100 nM). Inhibition of phospho-signal transducer and activator of transcription (STAT)-3 significantly reduced OCT3/4 and β-catenin levels and MMP-9 activity, ultimately resulting in reduced invasion. These results suggest that the anticancer effects of oleandrin and odoroside A might be due to the inhibition of invasion through of phospho-STAT-3-mediated pathways that are involved in the regulation of invasion-related molecules.

## 1. Introduction

The sodium/potassium (Na^+^/K^+^)-ATPase pump (NKP) inhibitors are gaining interest as candidates for cancer treatment. NKP is expressed in various cells such as neurons and cardiomyocytes and is a transmembrane ion transporter that is essential for cell survival, growth, and differentiation [1]. NKP serves as a multifunctional signal transducer and integrator and is responsible for maintaining resting potential and regulating cellular volume, contractility, adhesion, inflammation, and apoptosis [2,3,4,5]. Interestingly, it has been reported that the aberrant expression and activity of the pump are involved in the progression of several types of aggressive cancers [6,7,8,9,10], and this pump has been linked to cancer onset, proliferation and metastasis [11]. NKP has four α subunits, three β subunits, and one γ subunit [12], and the α1 and α3 subunits of NKP are frequently overexpressed in colorectal cancer, glioblastomas, and breast cancer [13,14,15].

Therefore, Na^+^/K^+^-ATPase is a crucial target for the development of anticancer agents. In this respect, cardiac glycosides, which are NKP inhibitors [16,17], could be new therapeutic agents that inhibit cancer cell survival by altering the distribution of NKP in cancer versus normal cells. The anticancer potential of cardiac glycosides has recently been evaluated, and promising data have been generated. For example, some cardiac glycosides, such as digitoxin, digoxin, bufalin, and ouabain, inhibit cancer cell proliferation and block tumor growth by inducing immunogenic cell death [18]. Selected cardiac glycosides (such as digoxin) are undergoing clinical trials [19,20] in multiple types of cancer including non-small cell lung carcinoma, colorectal cancer, breast cancer, melanoma, prostate cancer, and pancreatic cancer.

In particular, oleandrin, the most abundant cardenolide from *Nerium oleander*, has been the subject of much more extensive research regarding the mechanism of its action in cancer treatment. Oleandrin is a lipid-soluble cardiac glycoside that can effectively inhibit the proliferation of various cancer cells and induce apoptosis [21,22]. In addition, oleandrin can also enhance the effectiveness of radiotherapy [23]. Various studies to elucidate the possible pathways underlying the anticancer activity of oleandrin have been conducted, and it has been suggested that oleandrin which is known as an NKP inhibitor inhibits cancer cell proliferation by decreasing NKP levels as well [24], mitochondrial injury caused by the generation of reactive oxygen species (ROS) [25], activation of caspase-3, leading to tumor cell apoptosis [23], or activation of the DNA damage checkpoint at the G2/M phase [26]. However, the detailed mechanisms of the antitumor activity of oleandrin are still not fully understood.

The signal transducer and activator of transcription (STAT) protein family plays a major role in cancer [27]. STATs regulate the transcription of target genes that control tumor cell proliferation and differentiation, as well as genes encoding proteins with major roles in conditioning the tumor microenvironment [28,29,30]. Constitutive activation of STAT-3 has been found in invasive and metastatic tumors [31,32]. The activation of Janus kinase (JAK)-2/STAT-3 signaling regulates the growth and maintenance of stem-like breast cancer cells (CD44^+^CD24^−^) [33]. In addition, STAT-3 activation has also been associated with the resistance of tumor cells to chemotherapeutic agents and γ radiation [34,35]. STAT-3 mediates these effects through its collaboration with various other transcription factors, including nuclear factor-κB, hypoxia-inducible factor-1 and peroxisome proliferator activated receptor-γ, and via the upregulation of antiapoptotic gene products. Thus, downregulation of STAT-3 can overcome chemoresistance and radioresistance. However, there have been no studies investigating the effects of cardiac glycosides, such oleandrin and odoroside A, on the STAT-3 pathway.

The narrow therapeutic index of clinically used cardiac glycosides due to their cardiovascular toxicity could limit their therapeutic use [36,37]. To overcome this problem, it is necessary to search for cardiac glycoside-derived compounds with the ability to inhibit the proliferation and metastasis of cancer cells without causing cardiovascular damage. In vitro studies have shown that, at nanomolar concentrations, cardiac glycosides are nontoxic to normal cells and may protect them from apoptosis or induce cell proliferation, while in cancer cells, these drugs block cell proliferation and induce cell death [11,22,38,39,40]. Rashan et al. [41] reported that monoglycosidic cardenolides, including oleandrin and odoroside A isolated from *Nerium oleander*, exhibit significant anticancer activity. Unlike those of oleandrin, the effects of odoroside A and their underlying mechanisms are not well known.

The conventional therapies for breast cancer patients include surgical removal of the tumor, drug treatment and radiation. However, each therapy has inherent limitations that lead to therapeutic resistance and disease recurrence, ultimately resulting in therapeutic failure. Especially, the radio-resistance of breast cancer cells remains a clinical problem to the maximum efficacy of radiotherapy. Thus, in this study, using highly metastatic MDA-MB-231 and radio-resistant MDA-MB-231 breast cancer cells which were established in our previous study, we compared the anticancer effect of odoroside A with those of oleandrin and explored the associated mechanisms. 

## 2. Results

### 2.1. Oleandrin and Odoroside A Showed Effective Anticancer Effects Both in MDA-MB-231 and Radiotherapy Resistant (RT-R)-MDA-MB-231 at the Nanomolar Concentrations

**Figure 1:**
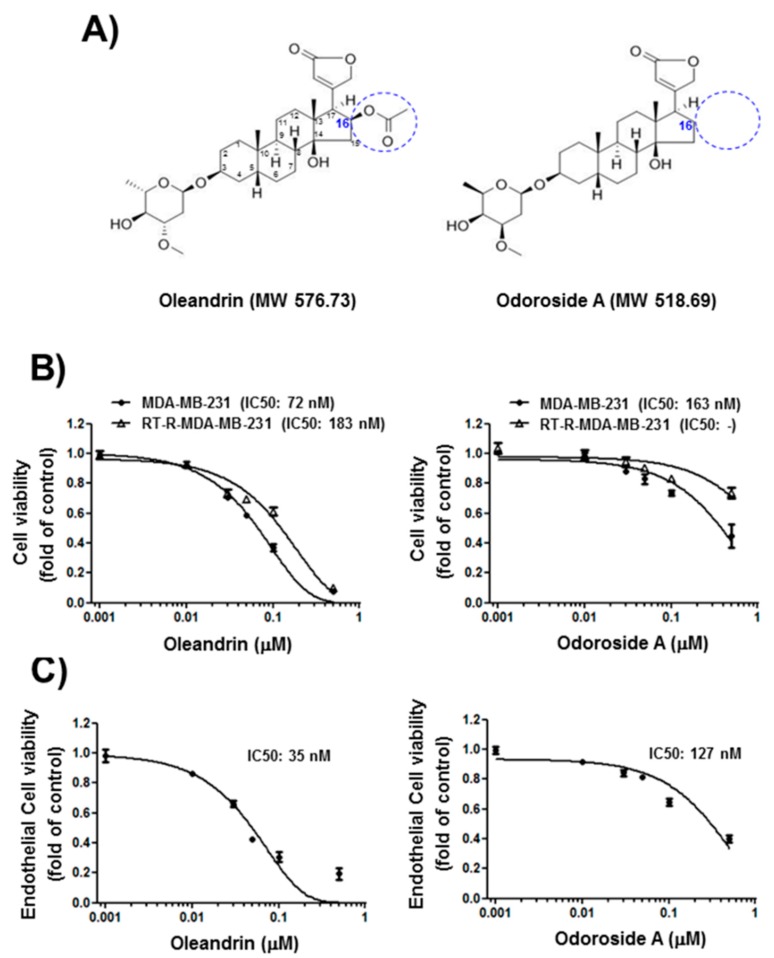
Chemical structure of oleandrin and odoroside A (**A**) and their effects on cell viability in MDA-MB-231 or RT-R-MDA-MB-231 cells (**B**) and ECs (**C**). (**B**,**C**) Cells were starved overnight and then treated with oleandrin and odoroside A at the indicated concentrations (1, 10, 30, 50, 100, and 500 nM). After 24 h, cell viability was determined by 3-(4,5-dimethylthiazol-2-yl)-2,5-diphenyltetrazolium bromide (MTT) assay. The values are expressed as the means ± SEM from three independent determinations. Blue circle in panel A shows the difference in structure between oleandrin and odoroside A.

**Figure 2:**
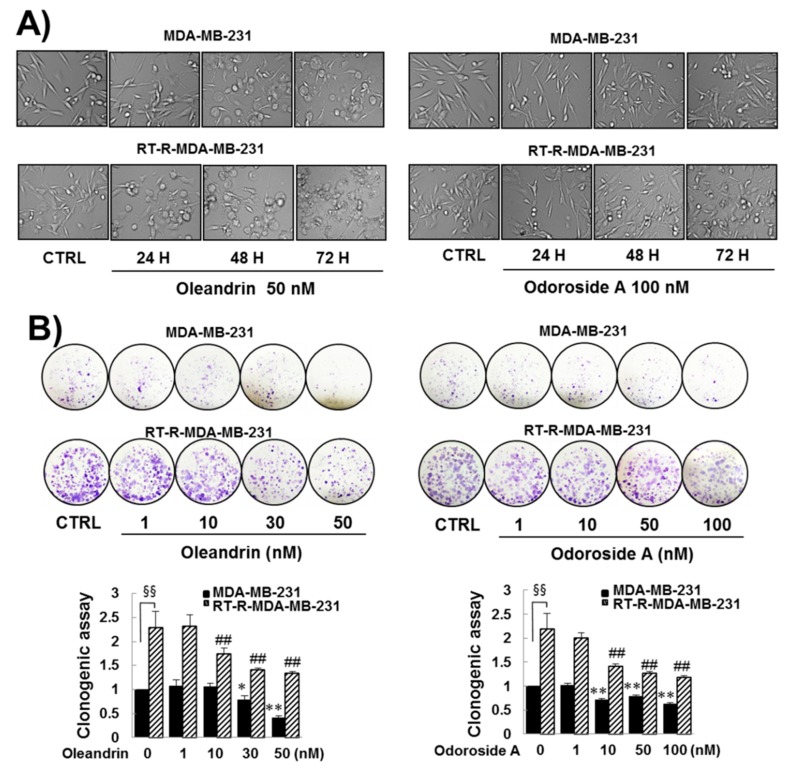
Effects of oleandrin and odoroside A on cell morphology (**A**) and colony formation (**B**) in MDA-MB-231 and RT-R-MDA-MB-231 cells. (**A**) Cells were treated with oleandrin (50 nM) and odoroside A (100 nM) for 24–72 h. Then, the morphologic changes of the cells were observed under a microscope (200×). (**B**) MDA-MB-231 and RT-MDA-MB-231 cells seeded in six-well plates (1000 cells/well) were treated with oleandrin and odoroside A at the indicated doses. After 24 h, the culture medium was discarded and replaced with fresh medium every 2–3 days. After 1–2 weeks, the cells were fixed, stained using crystal violet and counted. The values are expressed as the means ± SEM from three independent determinations. * *p* < 0.05, ** *p* < 0.01 compared with the control group of MDA-MB-231 cells; ^##^ *p* < 0.01 compared with control of RT-R-MDA-MB-231 cells. ^§§^ *p* < 0.01 between MDA-MB-231 and RT-R-MDA-MB-231 cells.

First, we determined the effects of oleandrin and odoroside A on the cell viability of breast cancer cells and endothelial cells (ECs) by MTT assay. The results revealed that oleandrin and odoroside A dose-dependently reduced the cell viability of breast cancer cells and ECs at nanomolar concentrations (Figure 1B,C). Interestingly, odoroside A seemed to be less toxic to ECs (IC50: 127 nM) and breast cancer cells MDA-MB-231 (IC50: 183 nM) and RT-R-MDA-MB-231 (IC50: noncalculated in these doses setting) than oleandrin (IC50: 35 nM in ECs, 72 nM in MDA-MB-231, 183 nM in RT-R-MDA-MB-231, respectively). Since we were more interested in the anti-invasive and antimetastatic effects of the drug candidates than their cytotoxicity due to the fact that cancer metastasis, rather than primary tumors themselves, is responsible for the majority of cancer deaths, we chose concentrations below the IC50s (50 nM for oleandrin and 100 nM for odoroside A) for the next experiments. Accordingly, we examined the time-dependent effects of oleandrin and odoroside A on the cell morphology of MDA-MB-231 and RT-R-MDA-MB-231 cells at 50 and 100 nM, respectively. Figure 2A shows that oleandrin (50 nM) and odoroside A (100 nM) induced morphologic changes in MDA-MB-231 cells and RT-R-MDA-MB-231 cells in a time-dependent manner (24, 48, and 72 h). Remarkably, both cell lines treated with oleandrin showed morphological changes, such as cell shrinkage, blebbing and disorganization, at 48 and 72 h (Figure 2A). In addition, oleandrin, and odoroside A significantly decreased colony formation in both cell lines in a dose-dependent manner (1, 10, 30, and 50 nM) (Figure 2B). These results suggest that oleandrin and odoroside A have anticancer effects by reducing clonogenicity at doses lower than their IC50s for cell viability; moreover, odoroside A is less toxic than oleandrin but has similar anticancer effects.

### 2.2. Oleandrin and Odoroside A Significantly Inhibited the Invasion of MDA-MB-231 and RT-R-MDA-MB-231 Cells at Lower Doses than IC50 of Cell Viability

**Figure 3:**
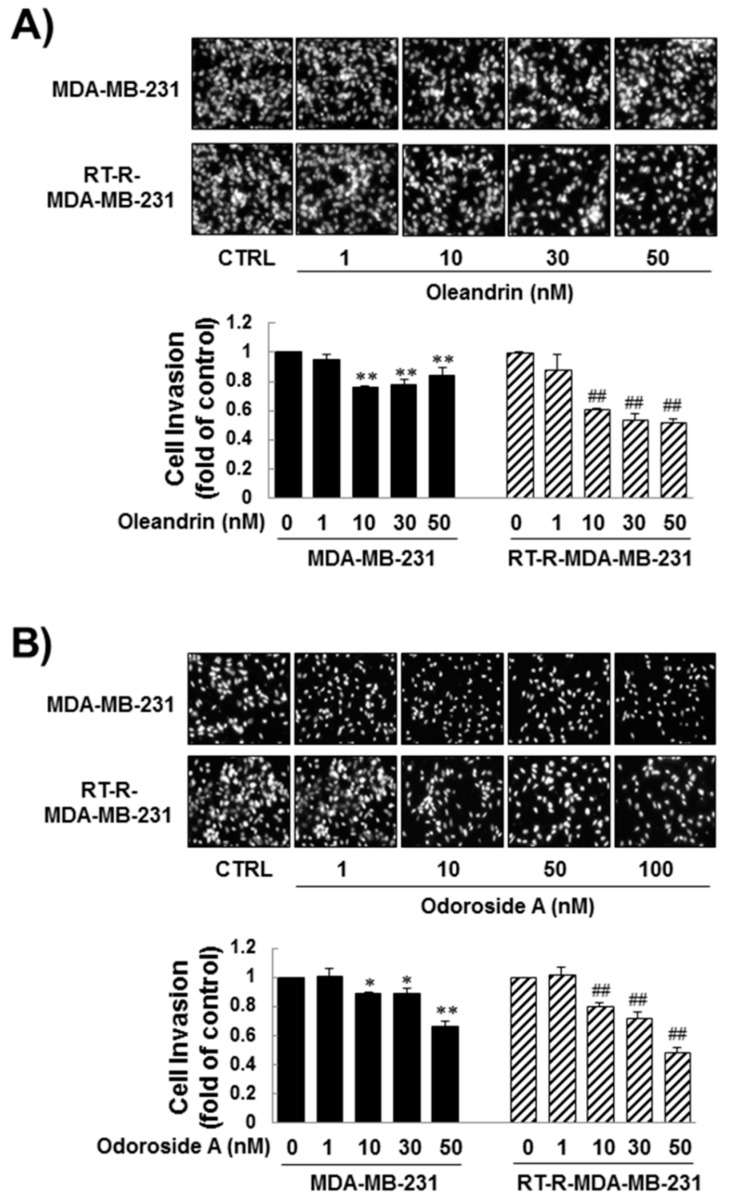
Inhibitory effect of oleandrin and odoroside A on the invasion of MDA-MB-231 and RT-MDA-MB-231 cells. MDA-MB-231 and RT-MDA-MB-231 cells were treated with oleandrin (**A**) and odoroside A (**B**) at the indicated concentrations for 24 h, and then the cells were collected, added to ECs-Matrigel-coated insert wells and incubated overnight (for 16 h) at 37 °C. The cells that had invaded across the membrane were stained with 4’,6-diamidino-2-phenylindole (DAPI) and counted under a fluorescence microscope (×200). The values are expressed as the means ± SEM from three independent determinations. * *p* < 0.05, ** *p* < 0.01 compared with the control group of MDA-MB-231 cells; ^##^ *p* < 0.01 compared with the control group of RT-R-MDA-MB-231 cells.

Next, we determined the effects of oleandrin and odoroside A on the invasion of MDA-MB-231 and RT-R-MDA-MB-231 cells through endothelial cells (ECs). Matrigel invasion assays revealed that oleandrin and odoroside A significantly inhibited cell invasion in a dose-dependent manner. Invasion was significantly inhibited by 1–50 nM oleandrin and by 1–100 nM odoroside A, which are doses lower than the IC50 values of these drugs for cell viability (Figure 3A,B).

### 2.3. Oleandrin and Odoroside A Inhibited Octamer-Binding Transcription Factor 3/4 (OCT3/4) and β-Catenin Expression and Reduced Matrix Metalloproteinase-9 (MMP-9) Secretion in MDA-MB-231 and RT-R-MDA-MB-231 Cells

**Figure 4:**
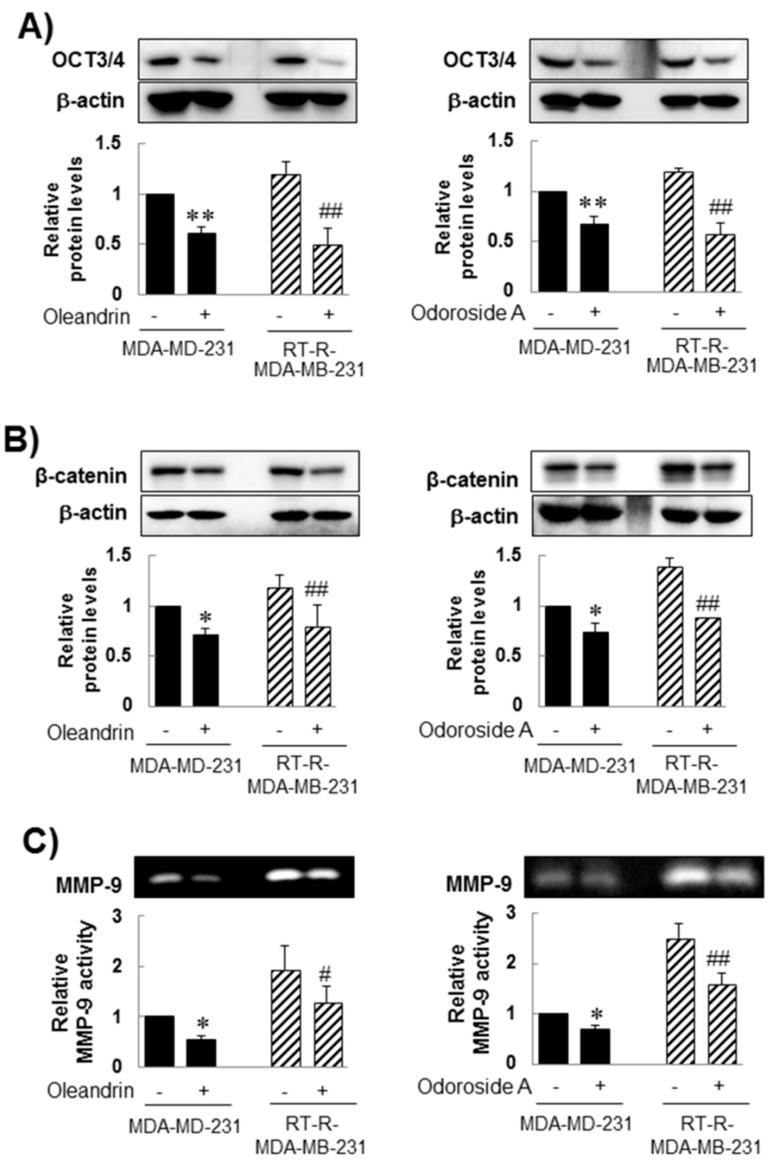
Inhibitory effect of oleandrin and odoroside A on OCT3/4 (**A**) and β-catenin expression (**B**) and MMP-9 secretion (**C**). Cells were treated with oleandrin (50 nM) and odoroside A (100 nM) for 24 h. After treatment, OCT3/4, β-catenin and β-actin protein levels were determined from the cell lysates by western blot analysis (**A**,**B**), and the gelatinolytic activity of MMP-9 was determined from the media by gelatin zymography as described in the Methods (**C**). The values are expressed as the means ± SEM from three independent determinations. * *p* < 0.05, ** *p* < 0.01 compared with the control group of MDA-MB-231 cells; ^##^ *p* < 0.01 compared with the control group of RT-R-MDA-MB-231 cells.

It has been reported that cancer stem cells (CSCs) exist in tumors and can be a cause of tumor resistance to chemotherapy and irradiation, contributing to cancer metastasis and cancer recurrence [42,43]. Our previous study reported higher expression of CSC markers and epithelial-mesenchymal transition (EMT) markers in RT-R-MDA-MB-231 cells than in MDA-MB-231 cells [44]. Thus, we investigated the effect of oleandrin and odoroside A on CSC marker levels and EMT protein levels. Western blot analysis showed that MDA-MB-231 and RT-R-MDA-MB-231 cells showed high protein levels of OCT3/4, a CSC marker, and β-catenin, an EMT protein. In addition, as expected, RT-R-MDA-MB-231 cells showed slightly higher expression levels of OCT3/4 and β-catenin than MDA-MB-231 cells, and the expression of these proteins was significantly inhibited by treatment with oleandrin (50 nM) and odoroside A (100 nM) for 24 h (Figure 4A,B). In addition, treatment with oleandrin (50 nM) and odoroside A (100 nM) for 24 h also effectively reduced MMP-9 activity in both MDA-MB-231 and RT-R-MDA-MB-231 cells (Figure 4C).

### 2.4. Oleandrin and Odoroside A Down-Regulated STAT-3 Phosphorylation Which Was Induced in MDA-MB-231 and RT-R-MDA-MB-231

**Figure 5:**
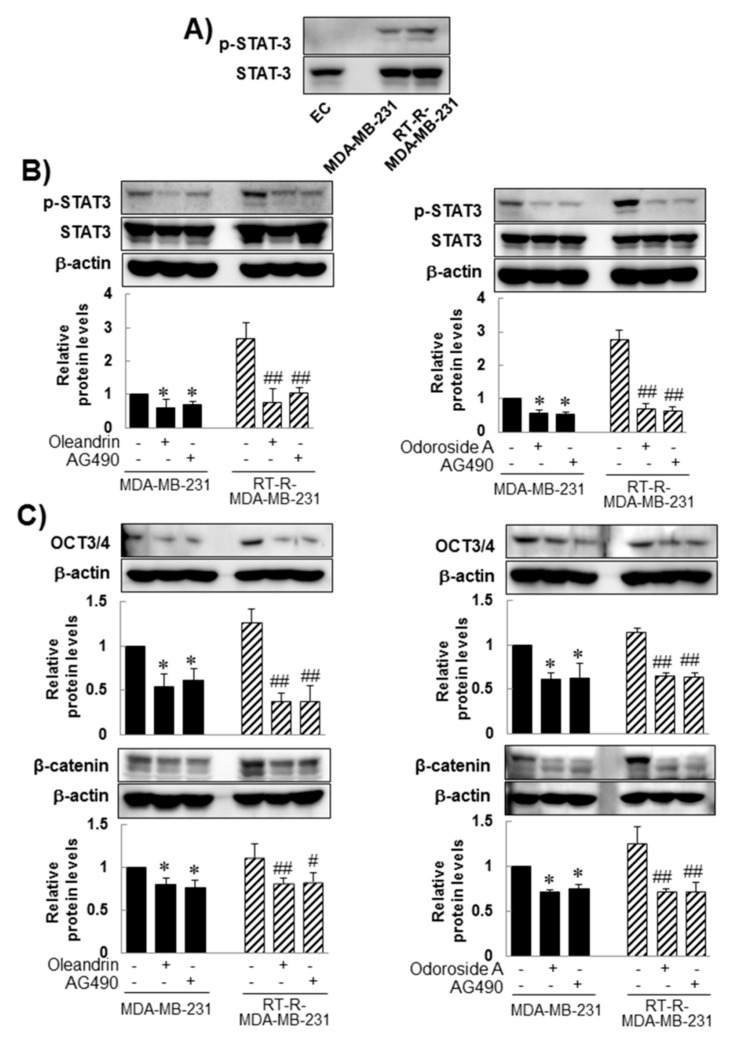

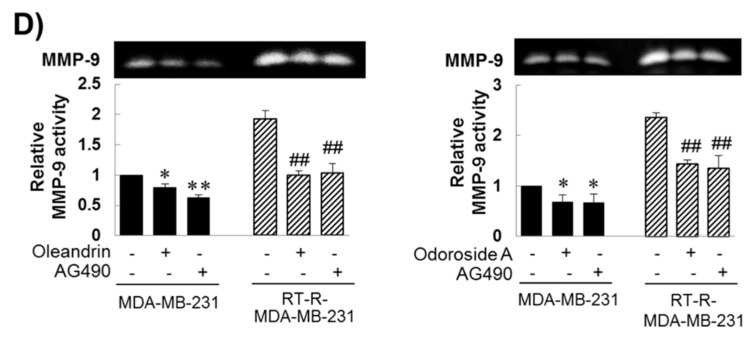
Inhibitory effects of oleandrin and odoroside A on Oct3/4, β-catenin, and MMP-9 through the downregulation of STAT-3 phosphorylation. (**A**) Phospho-STAT-3 and total STAT-3 protein levels were detected in the cell lysates of ECs, MDA-MB-231 cells and RT-MDA-MB-231 cells by Western blot analysis. (**B**–**E**) MDA-MB-231 and RT-R-MDA-MB-231 cells were treated with oleandrin (50 nM), odoroside A (100 nM) and AG490 (10 μM; a phospho-STAT-3 inhibitor) (**B**–**D**) for 24 h. After treatment, phospho-STAT-3 (**B**), OCT3/4 and β-catenin (**C**) were determined by Western blot analysis, and MMP-9 activity was determined (**D**). The values are expressed as the means ± SEM from three independent determinations. * *p* < 0.05, ** *p* < 0.01 compared with the MDA-MB-231 control group; ^#^ *p* < 0.05, ^##^ *p* < 0.01 compared with the RT-R-MDA control group.

**Figure 6:**
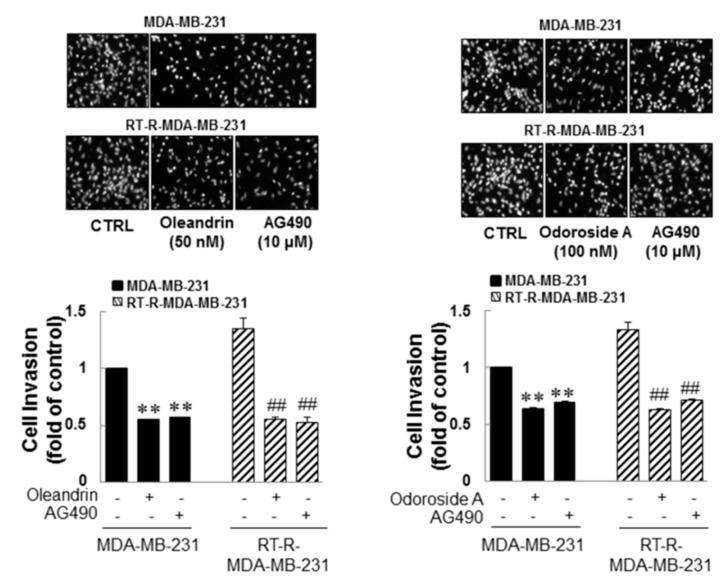
Anticancer effects of oleandrin and odoroside A in MDA-MB-231 and RT-MDA-MB-231 cells through the downregulation of phospho-STAT-3. MDA-MB-231 and RT-MDA-MB-231 cells were treated as described in Figure 5, and then the cells were collected and added to ECs-Matrigel-coated insert wells. The cells were incubated overnight (for 16 h) at 37 °C, and then the cells that had invaded across the membrane were stained with DAPI and counted as described in Figure 3 (×200 field image). The values are expressed as the means ± SEM from three independent determinations. ** *p* < 0.01 compared with the MDA-MB-231 control group; ^##^ *p* < 0.01 compared with the RT-R-MDA-MB-231 control group.

As mentioned in the Introduction, several studies have demonstrated that constitutive activation of STAT-3 occurs in a wide variety of tumors, including breast cancer, and participates in multiple cellular processes as well as in tumorigenesis. Thus, downregulation of STAT-3 has been suggested to overcome chemoresistance and radioresistance. Therefore, in this study, we investigated whether the anticancer effects of oleandrin and odoroside A in MDA-MB-231 and RT-R-MDA-MB-231 cells were mediated by modulation of the STAT-3 signaling pathway. Induced levels of phospho-STAT-3 in MDA-MB-231 and RT-R-MDA-MB-231 cells, but not those in ECs, (Figure 5A) were significantly reduced by treatment with oleandrin (50 nM) and odoroside A (100 nM) for 24 h, as shown in Figure 5B. The inhibitory effects of oleandrin (50 nM) and odoroside A (100 nM) on phospho-STAT-3 were the same as those of AG490 (a specific STAT-3 inhibitor). Moreover, AG490 significantly reduced the levels of OCT3/4 and β-catenin and the activity of MMP 9 in MDA-MB-231 and RT-R-MDA-MB-231 cells, effects that were similar to the inhibitory effects of oleandrin and odoroside A. Lastly, Figure 6 showed that STAT-3 inhibition by AG490 exhibited a similar effect of oleandrin (50 nM) and odoroside A (100 nM) on breast cancer cell invasion, indicating that the inhibition of STAT-3 phosphorylation by oleandrin and odoroside A mediates inhibition of breast cancer cell invasion.

## 3. Discussion

Various natural product-derived drugs play an important role in the oncology field, such as the vinca alkaloids vinblastine and vincristine isolated from *Catharanthus roseus* L. (Apocynaceae) and paclitaxel obtained from *Taxus brevifolia* Nutt. (Taxaceae). Among natural products, cardiac glycosides, which are traditionally used in the treatment of congestive heart failure and some arrhythmias because they inhibit NKP, have been attracting researchers’ attention for their cytotoxic, antitumor, and anticancer potential [37,40,45]. However, although promising data have shown them to be potential anticancer drugs, cardiac glycosides have a narrow therapeutic index due to their cardiovascular toxicity, which has become a barrier for their therapeutic use [36,37]. Some in vitro studies have shown that cardiac glycosides at nanomolar concentrations are nontoxic to normal cells but exhibit anticancer effects in cancer cells [11,26,38,39,40]. Thus, in this study, we aimed to screen cardiac glycosides that were ideally nontoxic or that has an acceptable toxicity to normal cells compared to their beneficial anticancer effect and then to investigate the anticancer effects of the candidates and their underlying mechanisms at safe dose ranges in highly metastatic MDA-MB-231 breast cancer cells and MDA-MB-231-derived radioresistant RT-R-MDA-MB-231 cells.

From preliminary experiments, we found that extract from *Nerium oleander*, more than any other natural products tested, was effective in inhibiting breast cancer cell invasion. Then, we proceeded to perform further experiments with oleandrin and odoroside A, which are known as active components of *Nerium oleander*. The results showed that oleandrin and odoroside A significantly decreased the colony formation of both MDA-MB-231 and RT-R-MDA-MB-231 cells at nanomolar ranges (Figure 2C). Moreover, oleandrin and odoroside A significantly inhibited the invasion of both MDA-MB-231 and RT-R-MDA-MB-231 cells through EC-Matrigel-coated transwell membranes (Figure 3) by downregulating the EMT-related protein β-catenin and the secretion of MMP-9 at 50 and 100 nM, respectively (Figure 4B,C). In addition, at the same doses, oleandrin and odoroside A effectively reduced the levels of the cancer stem cell marker OCT3/4 (Figure 4A). Finally, we found elevated levels of phospho-STAT-3 in MDA-MB-231 and RT-R-MDA-MB-231 cells that were higher in RT-R-MDA-MB-231 cells than in MDA-MB-231 cells (Figure 5A), and these levels were significantly decreased by oleandrin (50 nM) and odoroside A (100 nM) (Figure 5B). Inhibition of phospho-STAT-3 significantly reduced the expression of OCT3/4 and the activity of MMP-9, ultimately resulting in reduced invasion (Figure 5C,D and Figure 6).

Cardenolides are steroidal compounds characterized by the presence of a five-membered unsaturated lactone ring at C17 that exhibits cardiotonic activity. Cardenolides, together with the bufadienolides, which contain an α-pyrone ring, compose the group of so-called cardiac glycosides. Arai et al. [46] compared the anticancer activity of various compounds isolated from *Nerium oleander* and reported that the sugar moiety at C3 is important for anticancer activity. In addition, when a double bond at C16 and C17 was introduced, the activity decreased, and the OH group at C8 also reduced the activity. The difference in structure between oleandrin and odoroside A involves the presence or absence of an acetoxy group at C16, which is not important in conferring significant anticancer activity. According to our results, odoroside A, which has no acetoxy group at C16, shows reduced toxicity to normal ECs as well as to breast cancer cells, such as MDA-MB-231 and RT-R-MDA-MB-231 cells, at nanomolar ranges compared to oleandrin.

During past decades, it has been reported that oleandrin may be a potential antitumor agent, as it can effectively inhibit the proliferation of various cancer cells and induce apoptosis [21,22]. Additionally, oleandrin can enhance the effectiveness of radiotherapy [23]. The suggested mechanisms of oleandrin-mediated inhibition of tumor cell proliferation are as follows: alteration of membrane fluidity [47,48]; reduction of the activation of the nuclear transcription factors nuclear factor kappa B (NF-κB), c-Jun N-terminal kinase (JNK) and activator protein-1 (AP-1) [21,49]; elevation of intracellular calcium [22,36] and reactive oxygen species (ROS) production, oxidative injury and mitochondrial injury [22,25,26]; inhibition of fibroblast growth factor-2 (FGF-2) [48]; and weakening of the regulation of IL-8 receptors [50]. Cardenolides, as effective inhibitors of NKP, may regulate other signaling pathways. They can modulate the mitogen-activated protein kinase (MAPK)/extracellular signal-regulated kinase (ERK) pathway for antiproliferation by inhibiting NKP [51]. In addition, they also seem to induce autophagy in cancer cells by inhibiting hypoxia-inducible factor 1alpha (HIF1α), mammalian target of rapamycin (mTOR), and ERK1/2 [52,53,54]. However, the anticancer effects and the related mechanisms are not well understood, even though they may include one or more of those mentioned above for oleandrin.

This study provides important insights into the mechanisms of oleandrin and odoroside A; cardiac glycosides are anticancer agents that target the STAT-3 pathway. Aberrant activation of STATs, especially STAT-3, contributes to tumor progression at several levels. STATs regulate the transcription of target genes controlling tumor cell proliferation and differentiation, as well as genes encoding proteins with major roles in conditioning the tumor microenvironment, for instance, those involved in angiogenesis and the recruitment of immune cells [27,30]. In breast cancer, activation of STAT-3 and STAT-5 is frequently observed in cancer cells, with STAT-3 often activated in invasive and metastatic tumors [31,32]. Seven STAT proteins have been identified, and of these, STATs 2, 4, and 6 are activated specifically by a small subset of cytokines (IFNα, IL-6, 12, and 13, respectively). In contrast, STATs 1, 3, 5a, and 5b can be activated not only by a large array of cytokines but also by growth factors (EGF, PDGF, insulin, IGF-1, and others) and some G-protein coupled receptor agonists [55,56,57]. Recent studies have focused on the potential role of STATs (1, 3, 5a, and 5b) in oncogenic pathways [58]. Many STAT-3-regulated genes are involved in prosurvival signaling and the self-renewal of CSCs [59,60]. Increased STAT-3 activity has also been linked to the development of chemoresistance in triple-negative breast cancer cells (TNBCs) [61] and has been associated with metastasis promotion in TNBC [62].

Src homology region 2 domain-containing phosphatase 1 (SHP-1), a nonreceptor protein tyrosine phosphatase, is one of the negative regulators of phospho-STAT-3 [63]. In our preliminary data, oleandrin and odoroside A induced SHP-1 expression, which might result in the inhibition of STAT-3 phosphorylation (Supplemental Figure S1). Lee et al. [62] also reported that the herbal compound penta-*O*-galloyl-β-d-glycose (PGG) inhibited the JAK-1/STAT-3 axis through the induction of SHP-1 and ultimately suppressed TNBC growth and metastasis.

As mentioned in the Introduction, the α1 and α3 subunits of NKP are frequently overexpressed in colorectal cancer, glioblastomas and breast cancer [13,14,15], and thus far, it has been the dominant view that the aberrant overexpression and activity of the NKA α1 subunit is related to the progression of various cancers, including breast cancer [17]. The expression levels of NKA α1 in nine different breast cancer cell lines have been examined, and the relative NKA α1 expression in MDA-MB-231 and SKBR-3 cells, which are known as highly metastatic breast cancer cells, was the highest among all nine tested breast cancer cells [64], even though Salyer et al. [65] showed that the expression of α1, α3, and β3 was higher in MDA-MB-231 cells than in other breast cancer cells tested (T47D, MCF-7, and MDA-MB-453 cells). Further study to clarify the differential roles of α1 and α3 in breast cancer metastasis is needed, but targeting NKP-STAT-3 signaling may offer a novel therapeutic strategy to treat breast cancer.

## 4. Materials and Methods

### 4.1. Materials

Oleandrin and odoroside A were purchased from Sigma-Aldrich (St. Louis, MO, USA) (Figure 1A). Antibodies against OCT3/4, β-catenin and SHP-1 were purchased from Santa Cruz Biotechnology (Dallas, TX, USA), and antibodies against phospho-STAT-3 and STAT-3 were obtained from Cell Signaling Technology (Beverly, MA, USA). The BD Matrigel™ basement membrane matrix was supplied by BD Biosciences (San Diego, CA, USA). The enhanced chemiluminescence (ECL) Western blotting detection reagent was obtained from Bio-Rad (Hercules, CA, USA). All other reagents, including DAPI and an anti-β-actin antibody, were obtained from Sigma-Aldrich. 

### 4.2. Cell Culture

The human breast cancer cell line MDA-MB-231 was obtained from the Korea Cell Line Bank (Seoul, Korea), and the EA.hy926 human umbilical vascular endothelial cell line was originally purchased from the American Type Culture Collection (Manassas, VA, USA). RT-R-MDA-MB-231 cells were generated as described in a previous study [42]. Briefly, cells were irradiated with 2 Gy using a 6-MV photon beam produced by a linear accelerator (Clinac 21EX, Varian Medical Systems, Inc., Palo Alto, CA, USA) until a final dose of 50 Gy was achieved, which is a commonly used clinical regimen for radiotherapy in patients with breast cancer. The human breast cancer cell lines and EA.hy926 cells were cultured in Roswell Park Memorial Institute medium 1640 (RPMI 1640) and Dulbecco’s modification of Eagle medium (DMEM) (HyClone Laboratories, Logan, UT, USA), respectively, both of which were supplemented with 10% fetal bovine serum (FBS) (HyClone Laboratories), 100 IU/mL penicillin and 10 µg/mL streptomycin (HyClone Laboratories), and incubated at 37 °C in a humidified atmosphere containing 5% CO_2_ and 95% air.

### 4.3. Cell Viability Assay

Cells were seeded at a density of 1 × 10^4^ cells/well in 24-well plates. The cells were treated with oleandrin or odoroside A at concentrations of 1, 10, 30, 50, 100, and 500 nM at 37 °C for 24 h or were treated with 50 nM oleandrin or 100 nM odoroside A for 24 or 48 h. After the removal of media, the cells were washed with PBS and incubated for an additional 3 h in medium containing 0.1 mg/mL MTT. The supernatants were aspirated, and the formazan crystals were dissolved with 200 µL of 4 N HCl-isopropanol in each well. The optical density of the colored product was measured at 570 nm, as suggested by the manufacturer, using an Infinite 200 microplate reader (TECAN Austria GmbH, Grödig, Austria).

### 4.4. Colony Formation Assay

MDA-MB-213 cells or RT-R-MDA-MB-231 cells were seeded in six-well plates (1 × 10^3^ cells/well). Then, the cells were treated with oleandrin or odoroside A at the indicated doses at 37 °C for 24 h. After treatment, the culture medium was discarded and replaced every 2–3 days. After 1–2 weeks, the medium was discarded, and each well was washed with PBS. The colonies were fixed in 100% methanol for 10 min at room temperature and stained with 0.1% Giemsa staining solution for 30 min at room temperature, and then the visible colonies were counted.

### 4.5. Matrigel Invasion Assay

For the invasion assays, the upper chambers of the inserts were coated with 100 μL of Matrigel (1 mg/mL, BD Biosciences), and ECs (2 × 10^5^ cells) were added to the Matrigel-coated inserts. MDA-MB-231 cells and RT-R-MDA-MB-231 cells (2 × 10^5^ cells/insert) were treated with oleandrin or odoroside A at the indicated doses for 24 h and then added to the upper chambers in serum-free media, and 500 μL of RPMI-1640 with 10% FBS (HyClone Laboratories) was added to the lower chambers. The invasion chambers were incubated overnight (16 h) in a 37 °C cell culture incubator. The noninvasive cells that remained on the upper surface of the insert membranes were removed by scrubbing. The cells on the lower insert membranes were stained with DAPI, and the cells were counted under a fluorescence microscope (200× field image, Olympus, Tokyo, Japan).

### 4.6. Western Blot Analysis

Cells were harvested and lysed in RIPA buffer containing 50 mM Tris-HCl, pH 7.5, 150 mM NaCl, 1% NP-40, 0.1% SDS, 0.5% sodium deoxycholate and protease inhibitors. The samples were centrifuged at 16,000× *g* for 20 min at 4 °C, and the supernatants were collected for determination of the protein concentration using the Bradford method. Aliquots of 30–50 μg of protein were subjected to 8–10% sodium dodecyl sulfate-polyacrylamide gel electrophoresis (SDS-PAGE) for 2 h at 100 V. The separated proteins in the SDS-polyacrylamide gel were transferred onto Hybond-P+ polyvinylidene difluoride membranes (Amersham, Buckinghamshire, UK). The membranes were blocked with 5% nonfat milk in Tris-buffered saline containing 0.05% Tween-20 for 1 h at room temperature and then incubated with the following primary antibodies: anti-OCT3/4 (sc-9081, 1:1000, rabbit polyclonal IgG, Santa Cruz Biotechnology, Inc., Dallas, TX, USA), anti-β-catenin (sc-7199, 1:1000, rabbit polyclonal IgG, Santa Cruz Biotechnology), anti-phospho-STAT-3 (9131S 1:1000, Cell Signaling Technology, Danvers, MA, USA), anti-STAT-3 (4904S 1:1000, Cell Signaling Technology), and anti-SHP-1 (sc-7289 1:1000, Santa Cruz Biotechnology). The bound antibodies were detected with goat anti-rabbit IgG-horseradish peroxidase-conjugated secondary antibodies (sc-2054, 1:5000, Santa Cruz Biotechnology) and an ECL Western blotting detection reagent (Bio-Rad, Hercules, CA, USA).

### 4.7. Gelatin Zymography

MDA-MB-231 cells or RT-R-MDA-MB-231 cells (90% confluence) were treated with oleandrin or odoroside A at the indicated doses for 24 h, and then two milliliters of media was collected from cultured MDA-MB-231 cells or RT-R-MDA-MB-231 cells and concentrated 20-fold using protein concentrators (9K MWCO; Thermo Fisher Scientific, Inc., Waltham, MA, USA). Concentrated media containing the same amount of protein (40 μg/20 μL) were mixed with sample buffer (0.03% bromophenol blue, 0.4 M Tris-HCl pH 7.4, 20% glycerol, 5% SDS) and subjected to electrophoresis on 8% polyacrylamide gels containing 1 mg/mL gelatin. The gels were washed with renaturing buffer (2.5% Triton X-100) for 1 h and subsequently incubated for 24 h at 37 °C in developing buffer (50 mM Tris, 20 mM NaCl, 5 mM CaCl_2_, and 0.02% Brij35, pH 7.5). The gels were stained with 0.05% Coomassie Brilliant Blue R-250 and destained with 50% methanol and 10% acetic acid. Within the blue background, clear zones indicated MMP proteolytic activity.

### 4.8. Statistically Analyses

All results are representative of three independent experiments performed in triplicate. The statistical analysis was performed using SigmaPlot software (version 10.0; Systat Software, Inc., San Jose, CA, USA). The data were analyzed with two-tailed Student’s *t*-tests to compare two groups or with one-way analysis of variance with Scheffe’s post hoc test to compare mean values across multiple treatment groups. The data are presented as the means ± standard error.

## 5. Conclusions

Taken together, these results suggest that oleandrin and odoroside A have anticancer effects by inhibiting invasion/metastasis in both MDA-MB-231 and RT-R-MDA-MB-231 cells. In particular, odoroside A has less cytotoxicity to ECs than oleandrin but has similar anticancer effects. The anticancer effects of oleandrin and odoroside A might be due to the suppression of phospho-STAT-3-mediated pathways that are involved in the regulation of invasion-related molecules, such as cancer stem cell markers and EMT-related proteins.

## Supplementary Materials

Supplementary materials can be found at http://www.mdpi.com/1422-0067/19/11/3350/s1.

## Abbreviations

AP-1	Activator protein-1
CSC	Cancer stem cell
DAPI	4′,6-Diamidino-2-phenyindole, dilactate
DMEM	Dulbecco’s modification of Eagle medium
EC	Endothelial cell
ECL	Enhanced chemiluminescence
EMT	Epithelial-Mesenchymal Transition
ERK	Extracellular signal-regulated kinase
FBS	Fetal bovine serum
FGF-2	Fibroblast growth factor-2
HIF1α	Hypoxia-inducible factor 1alpha
IC50	Inhibitory concentration that decreases cell viability by 50%
JAK	Janus kinase
MAPK	Mitogen-activated protein kinase
MMP	Matrix metalloproteinase
mTOR	Mammalian target of rapamycin
MTT	3-(4,5-Dimethylthiazol-2-yl)-2,5-biphenyl tetrazolium bromide
NF-κB	Nuclear factor kappa B
NKP	Sodium/potassium (Na^+^/K^+^)-ATPase pump
OCT	octamer-binding transcription factor
ROS	Reactive oxygen species
RPMI	Roswell Park Memorial Institute medium
RT-R	Radioresistant
SDS-PAGE	Sodium dodecyl sulfate-polyacrylamide gel electrophoresis
SHP-1	Src homology region 2 domain-containing phosphatase 1
STAT	Signal transducer and activator of transcription
TNBC	Triple negative breast cancer
