# Supplementary figures and images for: Oleandrin and Its Derivative Odoroside A, Both Cardiac Glycosides, Exhibit Anticancer Effects by Inhibiting Invasion via Suppressing the STAT-3 Signaling Pathway
Source: Int J Mol Sci. 2018 Oct 26;19(11):3350. doi: 10.3390/ijms19113350 (PMC6274837; doi:10.3390/ijms19113350)

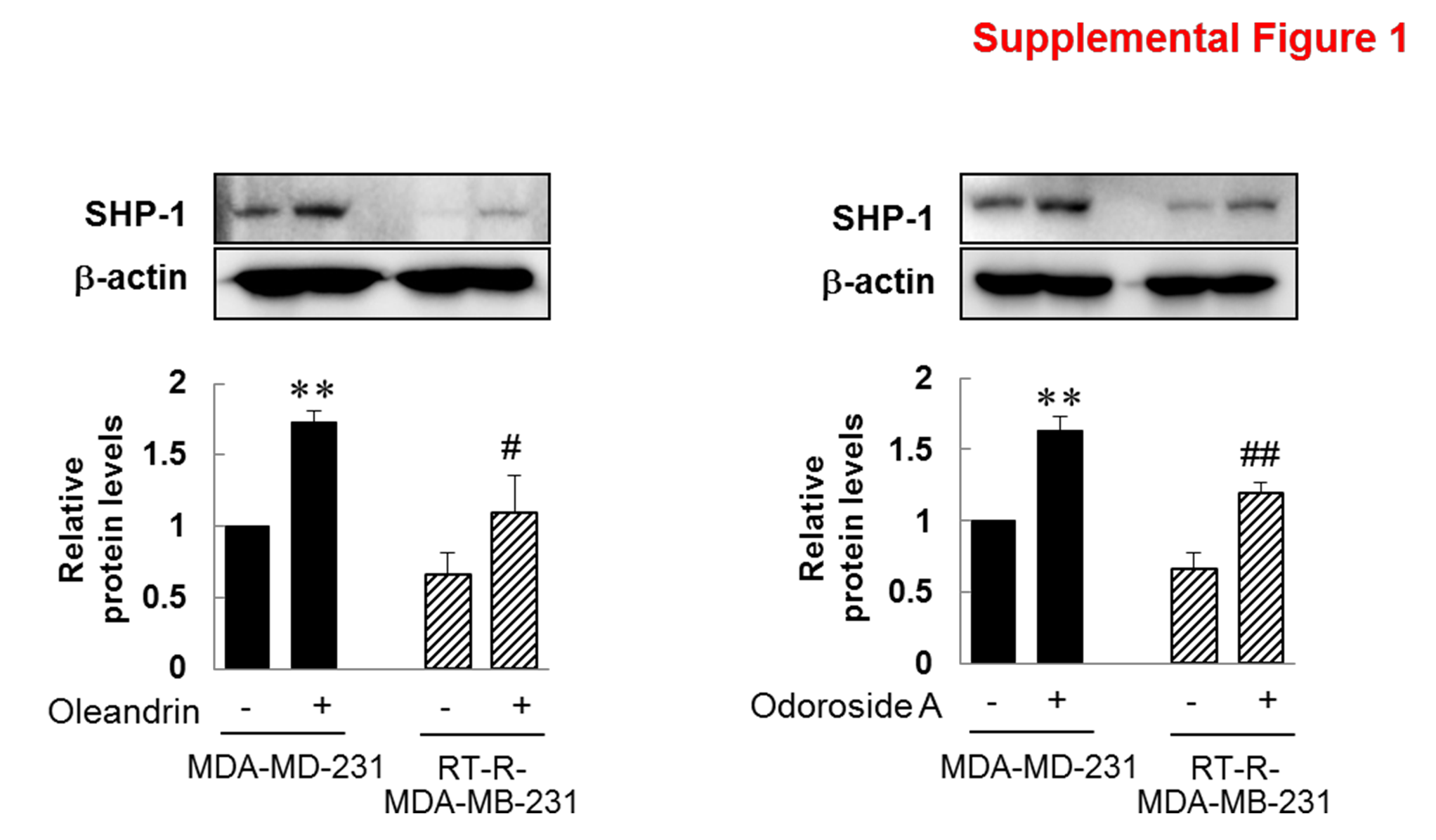

Supplement: Supplementary file 1 [file ijms-19-03350-s001.zip › ijms-361283-supplementary.tif]
